# Predicting RNA distance-based contact maps by integrated deep learning on physics-inferred secondary structure and evolutionary-derived mutational coupling

**DOI:** 10.1093/bioinformatics/btac421

**Published:** 2022-06-25

**Authors:** Jaswinder Singh, Kuldip Paliwal, Thomas Litfin, Jaspreet Singh, Yaoqi Zhou

**Affiliations:** Signal Processing Laboratory, School of Engineering and Built Environment, Griffith University, Brisbane, QLD 4111, Australia; Signal Processing Laboratory, School of Engineering and Built Environment, Griffith University, Brisbane, QLD 4111, Australia; Institute for Glycomics, Griffith University, Parklands Dr. Southport, QLD 4222, Australia; Signal Processing Laboratory, School of Engineering and Built Environment, Griffith University, Brisbane, QLD 4111, Australia; Institute for Glycomics, Griffith University, Parklands Dr. Southport, QLD 4222, Australia; Institute for Systems and Physical Biology, Shenzhen Bay Laboratory, Shenzhen 518055, China; Peking University Shenzhen Graduate School, Peking University, Shenzhen 518055, China

## Abstract

**Motivation:**

Recently, AlphaFold2 achieved high experimental accuracy for the majority of proteins in Critical Assessment of Structure Prediction (CASP 14). This raises the hope that one day, we may achieve the same feat for RNA structure prediction for those structured RNAs, which is as fundamentally and practically important similar to protein structure prediction. One major factor in the recent advancement of protein structure prediction is the highly accurate prediction of distance-based contact maps of proteins.

**Results:**

Here, we showed that by integrated deep learning with physics-inferred secondary structures, co-evolutionary information and multiple sequence-alignment sampling, we can achieve RNA contact-map prediction at a level of accuracy similar to that in protein contact-map prediction. More importantly, highly accurate prediction for top L long-range contacts can be assured for those RNAs with a high effective number of homologous sequences (*N*_eff_ > 50). The initial use of the predicted contact map as distance-based restraints confirmed its usefulness in 3D structure prediction.

**Availability and implementation:**

SPOT-RNA-2D is available as a web server at https://sparks-lab.org/server/spot-rna-2d/ and as a standalone program at https://github.com/jaswindersingh2/SPOT-RNA-2D.

**Supplementary information:**

Supplementary data are available at *Bioinformatics* online.

## 1 Introduction

3D structures of non-coding RNAs are the key for understanding their biological functions. Although these structures can be obtained by X-ray crystallography, nuclear magnetic resonance (NMR) or cryogenic electron microscopy, the low throughput and costly nature of these experimental techniques and the challenges associated with RNA structure determinations has led to fewer than 3% deposited structures in protein data bank (Rose *et al.*, 2017) containing RNAs and fewer than 0.01% of 18 million non-coding RNAs collected in RNAcentral (Consortium, 2020) with known structures. As a result, many computational and experimental methods have been developed for predicting or probing RNA secondary structure and base contacts (Cai *et al.*, 2020; Janssen and Giegerich, 2015; Lorenz *et al.*, 2011; Luo *et al.*, 2021; Reuter and Mathews, 2010; Solayman *et al.*, 2022) along with 1D to multi-dimensional structural probing data (Carlson *et al.*, 2018; Kubota *et al.*, 2015; Tinoco and Bustamante, 1999) to act as restraints for RNA 3D structure prediction (Watkins *et al.*, 2020; Zhang *et al.*, 2020b, 2020c).

Recently, computational prediction of RNA 3D structures has been improved by restraining distance-based contacts from direct coupling analysis analysis (DCA) (De Leonardis *et al.*, 2015a; Wang *et al.*, 2017a; Weinreb *et al.*, 2016). DCA restraints produced by these methods, however, were limited to <4000 families in the RFAM (Kalvari *et al.*, 2018) database, which provides multiple-sequence-alignment (MSA) manually curated according to experimentally determined secondary structures. Although this limitation was removed by the development of a pipeline named RNAcmap (Zhang *et al.*, 2021) that searches for and aligns with co-variant homologous sequences automatically, the accuracy of DCA predictors remains low, especially for those RNAs with few homologous sequences (Pucci *et al.*, 2020).

In the past few years, predicting distance-based contact maps of proteins has been improved significantly over DCA predictors by utilizing an ensemble of deep neural networks with improved evolutionary profiles and DCA results as an input (Hanson *et al.*, 2018; Li *et al.*, 2021; Wang *et al.*, 2017b; Yang *et al.*, 2020). Inspired by these studies, we developed a 2D deep-learning-based predictor SPOT-RNA (Singh *et al.*, 2019, 2021a) for secondary and tertiary base pairs and Sun et al. established the method for predicting distance-based contact maps [RNAContact (Sun *et al.*, 2021)]. RNAContact used evolutionary information; however, it neither took advantage of correlated mutations for deeper homology search nor used correlated mutations as input. Moreover, the method seems to perform poorly for the RNAs with few homologous sequences, which are the case for the majority of RNAs.

In this work, we improved the accuracy of predicting RNA distance-based contact map with improved co-evolutionary information from RNAcmap (Zhang *et al.*, 2021) and multiple sequence alignment sampling techniques used in trRosetta (Yang *et al.*, 2020) and AlphaFold (Senior *et al.*, 2020). Moreover, we used predicted secondary structures from a single-sequence-folding-based technique RNAfold as an input to improve the baseline performance for those RNAs without homologs. The new method, called SPOT-RNA-2D, substantially improves over existing methods in distance-based contact prediction, even at the single-sequence level.

## 2 Materials and methods

### 2.1 Datasets

We used the same benchmark datasets from our previous work on RNA backbone angle prediction (Singh *et al.*, 2021b) (SPOT-RNA-1D) for training, validation and testing. The SPOT-RNA-1D datasets consist of a training set (TR) of 286 RNA chains, a validation set (VL) of 30 RNA chains, and three test sets (TS1, TS2, TS3) of 63, 30 and 54 RNA chains, respectively, all prepared by using high-resolution RNA structures from PDB (Rose *et al.*, 2017).

More specifically, we downloaded all the high-resolution (<3.5 Å) X-ray structures from PDB on 3 October 2020. These RNA structures were split into individual chains using a *PDBParser* from Biopython (Cock *et al.*, 2009) and then sequences clustered using CD-HIT-EST (Fu *et al.*, 2012) at the lowest allowed identity cut-off of 80%. Non-clustered RNAs were kept for the validation and test sets while the remaining clustered RNAs for the training set.

As the 80% sequence identity cut-off may not be strict enough, therefore, we further used the BLAST-N (Altschul *et al.*, 1997) tool on non-clustered RNAs against training set and within themselves with a large e-value cut-off of 10. Any non-clustered sequence hits with the training set were removed from the training set, and any non-clustered sequence hits within themselves were also removed. After the CD-HIT-EST and BLAST-N filtering on non-clustered RNAs chains, we randomly split these RNAs chains into one validation (VL) and two test sets (TS1 and TS2).

Furthermore, we made VL and TS2 non-redundant even at the remote-homolog level from the other datasets (TR, TS1) and within themselves (VL, TS2). To achieve non-redundancy at the remote-homolog level, we build a covariance model for RNAs in VL and TS2. The covariance was built by first searching the query RNA (from VL, TS2) against NCBI’s database for homologs using BLAST-N. Then the multiple sequence alignment (MSA) of homologs along with consensus secondary structure (CSS) from 3D structural files was used to build the covariance model using the *cmbuild* program from the INFERNAL (Nawrocki and Eddy, 2013) tool. Finally, the covariance model of VL and TS2 RNAs was searched against the TR and TS1 using the *cmsearch* program from INFERNAL with an E-value cut-off of 0.1 for VL and 10 for TS2. Any hits of covariance models were removed from TR and TS1. Similarly, VL and TS2 were made non-redundant at the remote-homolog level within themselves. We used an E-value cut-off of 0.1 for VL to maintain a reasonable number of RNAs in the validation set (VL) and 10 for TS2 to make this test set as strict as possible for benchmarking. Tertiary structure predictions were evaluated on the TS2 set as it represents the most challenging set of targets for SPOT-RNA-2D.

The final X-ray dataset consists of 286, 30, 63 and 30 RNA chains for TR, VL, TS1 and TS2, respectively. The distribution of the datasets, such as the number of RNA chains in each set, median and maximum sequence lengths, type of base-pairs, the average number of distance-based contacts, is shown in Supplementary Table S1. We used the DSSR (Lu *et al.*, 2015) tool to extract different base pairs from 3D structural files.

These X-ray datasets were mapped to Rfam families (using the https://rfam.xfam.org/ website), to analyze the distribution and overlap of datasets in terms of Rfam families. As shown in Supplementary Table S2, few Rfam families are over-represented compared to others. This imbalance reflects the distribution of RNA 3D structures submitted to PDB. The utilization of CD-HIT-EST and BLAST-N to prepare test set TS1 remove Rfam family overlap of nearly 63% RNAs in comparison to training data as shown in Supplementary Table S2. The criterion of CD-HIT-EST and BLAST-N to prepare test sets has been used in past by RNAsol (Sun *et al.*, 2019), RNAsnap2 (Hanumanthappa *et al.*, 2021) and RNAContact (Sun *et al.*, 2021) methods. In addition, we further utilized INFERNAL to prepare datasets VL and TS2 by removing Rfam families overlap with training data nearly 100% except 1 RNA (2qus_A) from TS2. Moreover, we prepared another test set (TS-rfam) which consists of 69 RNAs from TS1 and TS2 that do not share any Rfam families overlap with training data. Performance on the test set TS-rfam is mentioned separately in Section 3.

During the preparation of the above datasets, we purposely kept RNA chains belong to the RNA-Puzzles (Cruz *et al.*, 2012; Miao *et al.*, 2015, 2017, 2020) in the test sets as many as possible because RNA-Puzzles RNAs are widely used for benchmarking RNA 3D models. There are 12 RNA chains belong to RNA-Puzzles, which was listed as a separate test set (named RNA-Puzzles) for benchmarking the predictors.

In addition to the above three test sets (TS1, TS2 and RNA-Puzzles), we prepared an additional test set (TS3) using NMR structures. To prepare TS3, we downloaded all the NMR structures (707) from the PDB (Rose *et al.*, 2017) on 5 April 2021. We made these RNAs non-redundant within themselves and from TR, VL, TS1 and TS2 using the exact same criterion as TS1 and obtained 54 RNA chains for benchmarking.

To obtain the distance-based contact labels, we defined two nucleotides in contact if the distance between the nearest-heavy atoms of these two nucleotides is <8 Å as done previously (De Leonardis *et al.*, 2015a; Weinreb *et al.*, 2016; Zhang *et al.*, 2021). Also, the Number of EFFective (*N*_eff_) for all the RNAs were obtained using GREMLIN tool, where *N*_eff_ is defined as the sum of weights after down-weighting each sequence by the number of neighbours above a pairwise sequence similarity cutoff of 0.8.

### 2.2 Input features

**Fig. 1.**
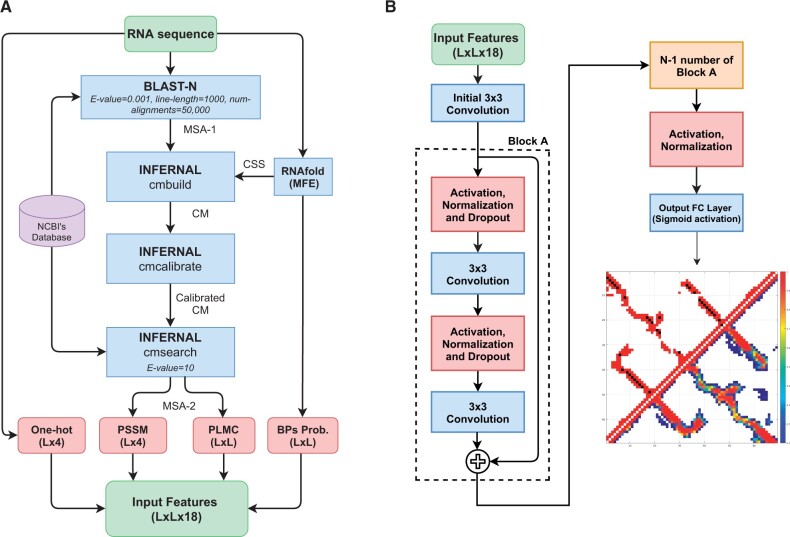
(**A**) Inputted 1D and 2D features used by the SPOT-RNA-2D; where MSA is multiple sequence alignment, CSS is predicted consensus secondary structure from RNAfold (MFE), CM is covariance model, One-hot is the one-hot encoding of the input sequence, PSSM is the position-specific scoring matrix, PLMC is pseudo-likelihood maximization coupling, BPs is predicted base-pairs probability from RNAfold (MFE), MFE is minimum free energy and L is the length of the RNA sequence. (**B**) The generalized deep neural network architecture of SPOT-RNA-2D

The input features for this work follow our previous base-pair predictor [SPOT-RNA2 (Singh *et al.*, 2021a)], which illustrated the importance of both single-sequence-based and evolutionary-profile-based features. Both types of features were used here for RNA distance-based contact map prediction, as shown in Figure 1A.

Single-sequence-based features include one-hot encoding of an input RNA sequence of size L×4, with (1,0,0,0), (0,1,0,0), (0,0,1,0) and (0,0,0,1) for four nucleotides (A, U, G and C), respectively, where L is the sequence length. Along with one-hot encoding, we used the predicted base-pair probability of size *L *×* L* from the single-sequence-based method RNAfold (Lorenz *et al.*, 2011). We preferred RNAfold over other predictors because it is one of the most accurate non-machine learning methods. We used a non-machine learning-based method to avoid any potential bias during performance evaluation on test sets.

Evolutionary-profile-based features include position-specific scoring matrix (PSSM) of size L×4 and 2D direct coupling analysis (DCA) information from PLMC (Balakrishnan *et al.*, 2011; Hopf *et al.*, 2017) of size *L *×* L*. PSSM and DCA features were extracted from RNAcmap (Zhang *et al.*, 2021) generated multiple-sequence-alignment (MSA-2) as shown in Figure 1A. RNAfold, rather than SPOT-RNA, was employed for generating consensus secondary structure (CSS) for RNAcmap.

All 1D features (one-hot encoding and PSSM, L×4) were converted into 2D features of size L×16 using the outer-concatenation function as described in RaptorX-Contact (Wang *et al.*, 2017b). Finally, 1D and 2D features were concatenated into a feature vector of size L×L×18 as an input to the deep neural network model, as shown in Figure 1B. These input features were standardized to have a zero mean with a unity variance according to the mean and the standard deviation in the training set before feeding into the deep neural network architecture.

### 2.3 Neural network architecture

The deep neural network architecture used in this work was inspired by our previous work SPOT-RNA (Singh *et al.*, 2019) and SPOT-RNA2 (Singh *et al.*, 2021a) for RNA secondary structure prediction. SPOT-RNA and SPOT-RNA2 utilized an ensemble of Residual Convolutional Neural Networks (He *et al.*, 2016) (ResNets). Similar to SPOT-RNAs, we used an ensemble of ResNets based on the generalized model architecture shown in Figure 1B.

The architecture of SPOT-RNA-2D (shown in Fig. 1B) consists of an initial convolutional layer with a kernel size of 3 × 3 and N_*F*_ filters followed by N_*A*_ ResNet blocks (Block-A). A single ResNet block consists of two pre-activated convolutional layers with a kernel size of 3 × 3 and N_*F*_ filters. The input to convolutional layers was also normalized by using layer normalization (Ba *et al.*, 2016). A dropout rate of 50% was used to avoid overfitting of model weights on training data.

Finally, a fully connected (FC) output layer with a single node and a sigmoid activation function was used. It predicts the upper triangular base-pair probability matrix of size L × L (as shown in Fig. 1B), where L is the length of the input sequence. A single threshold value was used to decide whether two nucleotides are in a distance-based contact or not. The value of the threshold was obtained by optimizing the F1-score on the validation set (VL).

We trained many models based on the architecture shown in Figure 1B by grid search for range of ResNets blocks (N_*A*_) and filters (N_*F*_). N_*A*_ was varied from 1 to 20 and N_*F*_ from 32 to 96 with a step increase of 1 and 8, respectively. Based on the performance of the validation set, we choose the four best models for the ensemble. Model architecture parameters for these four models are shown in Supplementary Table S3.

All the models were implemented using Google’s TensorFlow framework (Version-1.15) and trained using Nvidia GTX TITAN X graphics processing unit (GPU). For training, an Adam optimizer (Kingma and Ba, 2014) with a learning rate of 0.005 was used. A ELU function (Clevert *et al.*, 2015) was used to apply non-linear activation to the output of every layer except the output FC layer. Model hyper-parameters such as optimizers, activation functions and dropout rates were obtained from our previous work of RNA secondary structure prediction (Singh *et al.*, 2019, 2021a).

### 2.4 Tertiary structure modelling

RNA 3D structure models were generated with the *rna_denovo* application of the Rosetta molecular modelling suite. Inputs were generated from the FARFAR2 (Watkins *et al.*, 2020) RNA benchmark pipeline (available: https://github.com/DasLab/rna_benchmark). In all cases, secondary structure assignments [from RNAfold (Lorenz *et al.*, 2011) or DSSR (Lu *et al.*, 2015)] were used to pre-assemble canonical A-form helices. Pseudoknotted base pairs were removed with the *RemovePseudoknots* application from the RNAstructure package (Reuter and Mathews, 2010). Predicted tertiary contacts were then identified based on previously defined criteria (De Leonardis *et al.*, 2015b), which excludes local sequence neighbours (|i−j|<5) and contacts neighbouring assigned secondary structure pairs (k±{0,1,2},l±{0,1,2}). Constraints were selected based on a fixed probability threshold (0.3 and 0.4 for SPOT-RNA-2D and SPOT-RNA-2D-Single). The probability threshold was optimized by a coarse grid search (Supplementary Fig. S1) and the improvement of SPOT-RNA-2D is reasonably robust to the specific threshold. Ambiguous atom-pair constraints were applied to all combinations of heavy atoms such that only the minimum-energy atom-pair impacted the global energy during the minimization. Constraint energy terms used the long-range definition defined previously (De Leonardis *et al.*, 2015b) with an empirical weight of 10 (FADE -100 26 20 -20 20). Models with tertiary constraints were generated by fragment assembly since the full atom refinement was not found to improve model accuracy when using tertiary constraints (not shown). Baseline models were generated with the full FARFAR2 protocol. In this work we report model accuracy as the C3’ RMSD of the native structure with the top 1 low energy model from 200 simulations.

### 2.5 Performance evaluation

Similar to RNA secondary structure prediction, distance-based contact-map prediction is a binary classification problem. Thus, we used precision [PR=TP/(TP+FP)], sensitivity [SN=TP/(TP+FN)] and F1-score [F1=2(PR×SN)/(PR+SN)] for non-local contacts [|i−j|≥4] as a performance measure, where *TP*, *FP* and *FN* denotes true positives, false positives and false negatives, respectively, and *i* and *j* are the sequence positions of any two nucleotides in a sequence. To evaluate binary classification performance metrics, the predicted base-pair probability was converted into binary classification using a threshold value that optimize the F1-score on the validation set for SPOT-RNA-2D (0.46) and SPOT-RNA-2D-Single (0.50). For other methods, we used a threshold value that optimizes the F1 score on the test set TS1.

In addition, for an RNA of length L, we used a mean precision of top L/1, L/2, L/5 and L/10 long-range contacts [|i−j|≥24] for benchmarking different predictors, where *i* and *j* are the of sequence positions of any two nucleotides in a sequence. This definition of long-range contacts [|i−j|≥24] has been utilized in past by DIRECT (Jian *et al.*, 2019) and RNAContact (Sun *et al.*, 2021). Tertiary structure models were evaluated by superimposed C3’ RMSD with the native structure computed with Biopython (Cock *et al.*, 2009).

### 2.6 Methods comparison

We compared SPOT-RNA-2D with PLMC (Balakrishnan *et al.*, 2011; Hopf *et al.*, 2017), GREMLIN (Kamisetty *et al.*, 2013), mean-field direct coupling analysis [mfDCA (Morcos *et al.*, 2011; Schug *et al.*, 2009; Weigt *et al.*, 2009)] and pseudolikelihood maximization [plmDCA (Ekeberg *et al.*, 2013)] direct coupling analysis algorithms. We downloaded the standalone program of PLMC (https://github.com/debbiemarkslab/plmc), GREMLIN (https://github.com/sokrypton/GREMLIN_CPP), mfDCA and plmDCA [from pydca (Zerihun *et al.*, 2020) https://github.com/KIT-MBS/pydca] and ran them locally. We also compared SPOT-RNA-2D with the recently developed deep learning-based method RNAContact (Sun *et al.*, 2021). We used the RNAContact webserver (https://yanglab.nankai.edu.cn/RNAcontact/) to obtain predictions.

For comparison with RNA secondary structure prediction methods, we downloaded RNAfold (Lorenz *et al.*, 2011) (from Vienna package version 2.4.14, available at https://www.tbi.univie.ac.at/RNA/), SPOT-RNA (Singh *et al.*, 2019) (available at https://github.com/jaswindersingh2/SPOT-RNA), SPOT-RNA2 (Singh *et al.*, 2021a) (available at https://github.com/jaswindersingh2/SPOT-RNA2) and LinearPartition (Zhang *et al.*, 2020a) (available at https://github.com/LinearFold/LinearPartition) and run these predictors locally with default parameters.

## 3 Results

### 3.1 Feature contribution, MSA sampling and ensemble learning

SPOT-RNA-2D utilizes two single-sequence-based and two evolutionary-based input features for RNA distance-based contact map prediction, as shown in Figure 1A. Single-sequence-based features include one-hot encoding (1D) and predicted base-pair probability (2D) from RNAfold (Lorenz *et al.*, 2011) (MFE). Evolutionary-based features include position-specific scoring matrix (PSSM, 1D) and direct coupling analysis (Balakrishnan *et al.*, 2011; Hopf *et al.*, 2017) (DCA, 2D) from RNAcmap generated multiple-sequence-alignment (MSA-2).

Table 1 shows the effect of the different input features on distance contact prediction used by a baseline model (Model-0) with the architecture shown in Figure 1B. This model was trained using a training set TR, validated by a validation set VL and tested on three non-redundant test sets TS1, TS2 and TS-rfam, all from high-resolution X-ray structures (Singh *et al.*, 2021b). The test set TS1 is made non-redundant from TR, VL, TS2 and within itself using CD-HIT-EST (Fu *et al.*, 2012) (lowest identity cutoff of 0.8) followed by BLAST-N (Altschul *et al.*, 1997) (E-value = 10). The test set TS2 (a harder test set) further excludes remote structural homologs by searching the INFERNAL covariance model of TS2 sequences against TR, VL, TS2 and within itself. The test set TS-rfam was prepared from TS1 and TS2 by extracting 69 RNAs that do not overlap with any Rfam family in the training data.

**Table 1.** Performance of the individual models according to Matthews Correlation Coefficient (MCC), F1-score, precision and sensitivity on the validation set VL1, and two test sets TS1, TS2 and TS-rfam

Predictor	VL1				TS1				TS2				TS-rfam			
	MCC	F1	Precision	Sensitivity	MCC	F1	Precision	Sensitivity	MCC	F1	Precision	Sensitivity	MCC	F1	Precision	Sensitivity
Single Sequence (SS)	0.663	0.713	0.835	0.622	0.671	0.691	0.853	0.581	0.622	0.640	0.812	0.529	0.645	0.664	0.826	0.555
SS + RNAfold (SPOT-RNA-2D-Single)	0.710	0.756	**0.863**	0.672	0.713	0.733	0.872	0.633	0.713	0.727	**0.883**	0.618	0.734	0.750	**0.890**	0.647
SS + PSSM	0.684	0.736	0.830	0.661	0.719	0.748	0.826	0.683	0.670	0.696	0.797	0.618	0.676	0.704	0.797	0.631
SS + DCA (PLMC)	0.681	0.737	0.808	0.677	0.702	0.736	0.779	0.698	0.694	0.720	0.808	0.650	0.681	0.710	0.795	0.642
SS + RNAfold + PSSM + DCA	0.697	0.749	0.828	0.683	0.724	0.752	0.830	0.687	0.714	0.737	0.838	0.657	0.730	0.752	0.851	0.674
SS + RNAfold + PSSM + DCA + MSA Sampling	0.705	0.757	0.827	**0.698**	0.782	0.806	0.854	**0.763**	0.713	0.739	0.815	**0.675**	0.729	0.755	0.829	**0.693**
SS + RNAfold + PSSM + DCA + MSA Sampling + Ensemble (SPOT-RNA-2D)	**0.712**	**0.760**	0.845	0.691	**0.800**	**0.819**	**0.893**	0.757	**0.735**	**0.754**	0.870	0.665	**0.755**	**0.773**	0.888	0.684

*Note*: Bold indicates the best performance metric of a model.

As shown in Table 1, the one-hot encoding only achieves an F1-score of 0.71, 0.69, 0.64 and 0.66 or Matthews Correlation Coefficient (MCC) of 0.66, 0.67, 0.62 and 0.64 for VL, TS1, TS2 and TS-rfam, respectively. An overall similar performance among different sets, despite the difference in fractions of tertiary base pairs and number of effective homologous sequences (Supplementary Table S1), indicates the robustness of the training.

Adding base-pairing probabilities from RNAfold (MFE) improves the F1-score by more than 6%, 4%, 13% and 13% for VL, TS1, TS2 and TS-rfam, respectively. For the evolution-based information, adding 1D PSSM and 2D DCA from PLMC (Balakrishnan *et al.*, 2011; Hopf *et al.*, 2017) improves F1-score more than 3%, 6%, 8% and 6% for VL, TS1, TS2 and TS-rfam, respectively. Combining both single-sequence-based and evolutionary-based features further increases the performance on test sets TS1, TS2 and TS-rfam. Note the performance on VL seems to reach a limit without (one hot encoding + RNAfold only) or with evolutionary information (one hot encoding + RNAfold + PSSM + DCA), largely due to the very low *N*_eff_-value of VL (median *N*_eff_ = 2, Supplementary Table S1).

To further extract the evolution information, we performed MSA sampling during training that was found effective for protein structure prediction by Alphafold (Senior *et al.*, 2020) and trRosetta (Yang *et al.*, 2020). This was done by using 20 random samples of 50, 100, 200, 500, 1000 RNAs from the full MSA alignment (MSA-2 in Fig. 1A). We chose 20 random seeds because SPOT-RNA-2D models took about 20 epochs to converge. PSSM and DCA features were evaluated for these sampled MSA before training. As shown in Table 1, MSA sampling improved the performance of the baseline model for validation (VL) and two test sets (TS1 and TS2). The amount of performance improvement observed was most for the TS1 because of relatively higher *N*_eff_-value (median *N*_eff_ = 40) in TS1 than in VL (median *N*_eff_=2) and TS2 (median *N*_eff_=9).

Finally, we used ensemble learning by performing the average ensemble of the outputs of the best four models from many models trained using the generalized architecture shown in Figure 1B. As shown in Table 1, ensemble learning further improves for the performance for validation (VL), and three test sets (TS1, TS2 and TS-rfam). Supplementary Table S4 shows Rfam family-wise performance of SPOT-RNA-2D on 93 RNAs from TS1 and TS2 with families overlap with training data highlighted in color red. There is no obvious trend of higher performance on families that exists in training in comparison to families that do not exist in training data. This shows the robustness of trained model to generalize across Rfam families that are not in training data.

### 3.2 Comparison with other predictors

Two methods SPOT-RNA-2D and SPOT-RNA-2D-Single were developed with and without evolution information, respectively. They were compared to four existing direct coupling analysis (DCA) predictors (PLMC, mfDCA, plmDCA and GREMLIN) on two high-resolution (<3.5 Å) non-redundant test sets (TS1 and TS2) derived from PDB X-ray structures and one non-redundant test set (TS3) derived from PDB NMR structures. Another test set TS-rfam prepared from TS1 and TS2 by extracting 69 RNAs without any Rfam family overlap with training set (TR). Test sets TS1 and TS2 contain 12 RNA-Puzzles RNAs with results also shown separately. To further compare with the recently developed deep learning-based RNAContact (Sun *et al.*, 2021) without biases, we removed redundant sequences in TS1, TS2, TS3, RNA-Puzzles and TS-rfam to the RNAContact training set and led to 21, 9, 52, 7 and 20 RNAs, respectively. We also downloaded the test set TS80 prepared by RNAContact. After removing the sequence identity of TS80 with our training data (TR) using the same criterion as RNAContact, we obtained 10 RNAs in TS80. We compared to DCA predictors on full test sets and to RNAContact on reduced test sets.

Table 2 shows that RNAContact, SPOT-RNA-2D and SPOT-RNA-2D-Single (F1 > 0.58) are all substantially better than DCA predictors (F1 ∼ 0.3) for all test sets. SPOT-RNA-2D improves over RNAContact in F1-score by 18%, 3.5%, 32%, 22%, 16% and 8% on the reduced test sets TS1, TS2, TS3, RNA-Puzzles, TS80 and TS-rfam, respectively. In fact, even the single-sequence-based predictor (SPOT-RNA-2D-Single) improves over RNAContact in F1-score by 1-3% better on TS1 and TS2 and 6-30% better on the TS3, RNA-Puzzles, TS80 and TS-rfam.

**Table 2.** Performance comparison among different predictors on full test sets TS1 (63 RNAs), TS2 (30 RNAs), TS3 (54 RNAs), RNA-Puzzles (12 RNAs), TS80 (10 RNAs) and TS-rfam (69 RNAs) and reduced test sets TS1 (21 RNAs), TS2 (9 RNAs), TS3 (52 RNAs), RNA-Puzzles (7 RNAs), TS80 (10 RNAs) and TS-rfam (20 RNAs) after removing the sequences overlapping with RNAContact training data

	TS1			TS2			TS3			RNA-Puzzles			TS80			TS-rfam		
	F1	Precision	Sensitivity	F1	Precision	Sensitivity	F1	Precision	Sensitivity	F1	Precision	Sensitivity	F1	Precision	Sensitivity	F1	Precision	Sensitivity
Full test sets																		
GREMLIN	0.242	0.228	0.258	0.146	0.156	0.136	0.065	0.463	0.035	0.118	0.258	0.077	0.259	0.207	0.345	0.123	0.182	0.093
plmDCA	0.338	0.342	0.334	0.269	0.261	0.279	0.233	0.387	0.166	0.194	0.367	0.131	0.333	0.393	0.289	0.256	0.284	0.233
mfDCA	0.348	0.301	0.414	0.307	0.244	0.412	0.343	0.313	0.379	0.271	0.254	0.291	0.353	0.372	0.335	0.301	0.247	0.387
PLMC	0.375	0.478	0.308	0.295	0.348	0.256	0.169	0.560	0.099	0.181	0.555	0.108	0.395	0.538	0.312	0.254	0.381	0.191
SPOT-RNA-2D-Single	0.734	0.876	0.631	0.727	**0.878**	0.620	0.755	**0.942**	0.630	0.747	0.909	0.634	0.779	0.902	0.685	0.750	**0.890**	0.647
SPOT-RNA-2D	**0.819**	**0.893**	**0.757**	**0.754**	0.870	**0.665**	**0.768**	0.923	**0.657**	**0.793**	**0.921**	**0.696**	**0.853**	**0.909**	**0.803**	**0.773**	0.888	**0.684**
Reduced test sets																		
RNAContact	0.714	0.848	0.616	0.721	0.853	0.625	0.581	**0.968**	0.415	0.671	0.880	0.542	0.737	0.846	0.653	0.715	0.855	0.615
SPOT-RNA-2D-Single	0.736	0.871	0.637	0.727	**0.835**	0.644	0.753	0.941	0.627	0.766	0.930	0.651	0.779	0.902	0.685	0.763	**0.862**	0.684
SPOT-RNA-2D	**0.844**	**0.894**	**0.800**	**0.745**	0.801	**0.696**	**0.765**	0.921	**0.654**	**0.818**	**0.937**	**0.725**	**0.853**	**0.909**	**0.803**	**0.772**	0.857	**0.702**

*Note*: Bold indicates the predictor with the best performance.

Overall, test sets TS2 and TS3 are more difficult to predict compared to the other test sets (TS1, RNA-Puzzles and TS80) for all the predictors. This is mainly due to the relatively low *N*_eff_-value of TS2 (median *N*_eff_=9, see Supplementary Table S1) and TS3 (median *N*_eff_=4, see Supplementary Table S1). Unlike evolutionary-profile-based predictors, SPOT-RNA-2D-Single is more consistent irrespective of *N*_eff_-value, showing the usefulness of a single-sequence-based predictor where evolutionary information is not available.

**Fig. 2.**
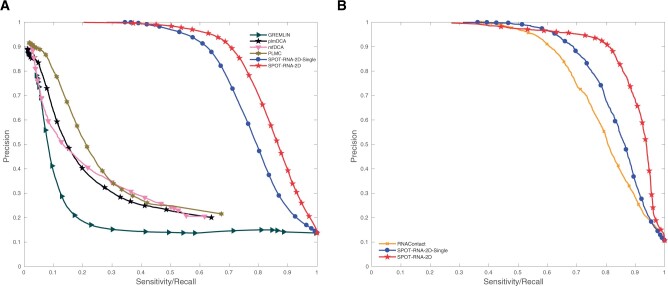
Precision–recall (PR) curves given by SPOT-RNA-2D and SPOT-RNA-2D-Single (**A**) along with four DCA predictors on 147 RNAs from three test sets TS1, TS2 and TS3, (**B**) further comparison with RNAContact on 82 RNAs from three reduced test sets TS1, TS2 and TS3 after removing the sequences overlapping with RNAContact training data

The large improvement of SPOT-RNA-2D over DCA methods can be further illustrated by the precision–recall curve (Fig. 2A) on combined 147 RNAs from three test sets TS1, TS2 and TS3. Both SPOT-RNA-2D and SPOT-RNA-2D-Single are significantly better than all DCA predictors for any threshold values. In Figure 2B, RNAContact was compared to SPOT-RNA-2D separately on the combined reduced test sets (82 RNAs). SPOT-RNA-2D-Single offers a large improvement over RNAContact with a 12% increase in the area under the precision–recall curve, and SPOT-RNA-2D provides an additional improvement of 11% over SPOT-RNA-2D-Single. Similar large improvements can be illustrated with the receiver operating characteristic curves shown in Supplementary Figure S2A and B.

**Fig. 3.**
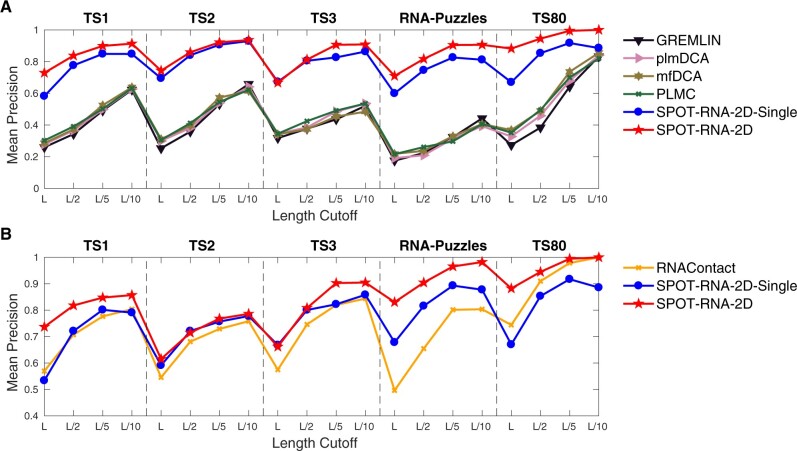
Mean precision of long-range contacts (i−j≥24) given by various methods as labelled (**A**) on full test sets TS1, TS2, TS3, RNA-Puzzles and TS80, (**B**) on reduced test sets TS1, TS2, TS3, RNA-Puzzles and TS80 after removing the sequences overlapping with RNAContact training data

Another way to measure the method performance in contact prediction is the fraction of true positions in top L, L/2, L/5 and L/10 predictions (Precision). The most important contacts are those contacts with large differences in sequence positions (long-range or non-local contacts). Figure 3A and B compares the precisions in long-range contacts [(i−j)≥24] given by DCA methods and RNAContact, respectively, with those by SPOT-RNA-2D and SPOT-RNA-2D-Single for five test sets (TS1, TS2, TS3, RNA-Puzzles and TS80). Again, a large improvement is observed.

**Fig. 4.**
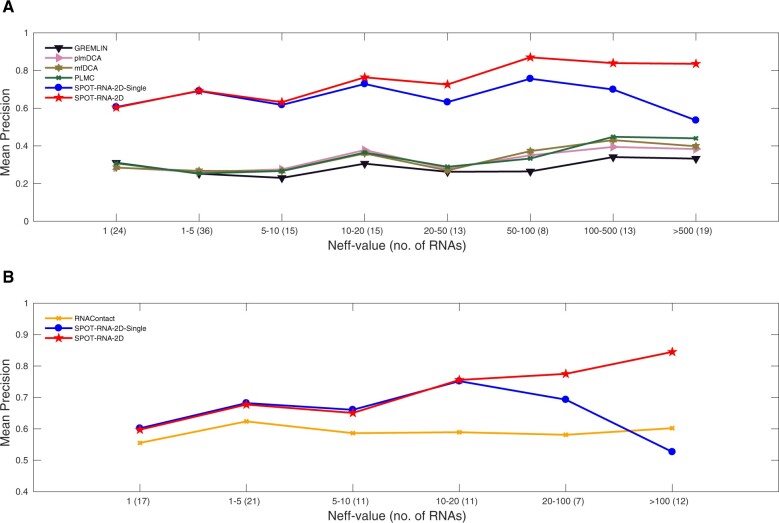
Mean precision of top L long-range contacts as a function of the number of effective homologous sequences N_eff_-value on (**A**) the combined full test sets TS1, TS2 and TS3 (**B**) the combined reduced test sets TS1, TS2 and TS3 after removing the sequences overlapping with RNAContact training data

To illustrate the impact of homologous sequences, Figure 4 shows the mean precision of long-range contacts as a function of *N*_eff_-value. The mean precisions for SPOT-RNA-2D and DCA predictors (Fig. 4A) increase with *N*_eff_-value with a large gap between them. Meanwhile, the performance of SPOT-RNA-2D-Single does not depend much on the *N*_eff_-value with similar performance to SPOT-RNA-2D for low *N*_eff_ RNAs, highlighting the robustness of the method performance. Figure 4B compares the performance of SPOT-RNA-2D and SPOT-RNA-2D-Single to that of RNAContact on the combined reduced test sets (TS1, TS2 and TS3.) The dependence of RNAContact on *N*_eff_ is surprisingly low, suggesting its ineffective use of evolutionary information. SPOT-RNA-2D yields a larger improvement over RNAContact for high *N*_eff_ RNAs. Even SPOT-RNA-2D-Single based on the single-sequence only outperforms RNAContact for all values of *N*_eff_, except at *N*_eff_>100. The similar performance at low *N*_eff_ between SPOT-RNA-2D and SPOT-RNA-2D-single indicates the effective use of evolutionary information by SPOT-RNA-2D without compromising performance for low *N*_eff_-value RNAs.

### 3.3 Base pairs and non-base pairs

Distance-based contacts involve paired bases and bases that are not paired but near each other. Base pairs include canonical, non-canonical, pseudoknots and multiples base pairs, all of which were annotated according to their native 3D structural files using the DSSR (Lu *et al.*, 2015) tool. Non-base pairs include all distance-based contacts, excluding the base pairs. As a reference, we also included representative RNA secondary structure predictors such as RNAfold (Lorenz *et al.*, 2011), LinearPartition (Zhang *et al.*, 2020a), SPOT-RNA (Singh *et al.*, 2019) and SPOT-RNA2 (Singh *et al.*, 2021a). Comparison to SPOT-RNA and SPOT-RNA2 was made on a reduced set after removing redundancy to their training sets.

F1-scores as a function of top L/n predictions given by various methods are shown in Supplementary Figure S3 for base pairs and Supplementary Figure S4 for non-base pairs. Results are further summarized in Table 3 by showing the mean highest F1-score given by each predictor in Supplementary Figures S3 or S4. The performance of all DCA predictors is in general poor for both base and non-base pairs. The standard folding-based secondary structure predictors RNAfold and LinearPartition improve over DCA methods for base pairs and comparable for non-base pairs. SPOT-RNA-2D has comparable mean F1-score for base pairs prediction in comparison to secondary structure predictors (RNAfold and LinearPartition), although it was not trained specifically for base pairs. More importantly, SPOT-RNA-2D is substantially more accurate for non-base pairs as non-base pairs comprise ∼85% of total distance-based contacts.

**Table 3.** Performance comparison in terms of mean F1-score for base pairs and non-local [(i−j)≥6] non-base pairs with DCA predictors (PLMC, mfDCA, plmDCA and GREMLIN) and secondary structure predictors (RNAfold and LinearPartition) on full test sets TS1 (63 RNAs), TS2 (30 RNAs) and TS3 (54 RNAs)

	TS1		TS2		TS3	
	Base pairs	Non-base pairs	Base pairs	Non-base pairs	Base pairs	Non-base pairs
Full test sets						
GREMLIN	0.414	0.170	0.365	0.176	0.305	0.229
plmDCA	0.445	0.200	0.401	0.196	0.341	0.240
mfDCA	0.454	0.203	0.417	0.202	0.348	0.240
PLMC	0.427	0.205	0.388	0.205	0.331	0.241
RNAfold	0.603	0.165	0.656	0.188	**0.776**	0.243
LinearPartition	0.623	0.166	**0.668**	0.179	0.764	0.258
SPOT-RNA-2D-Single	0.566	0.531	0.568	0.555	0.680	0.717
SPOT-RNA-2D	**0.690**	**0.642**	0.643	**0.590**	0.711	**0.730**
Reduced test sets						
SPOT-RNA	0.660	0.199	0.670	0.192	0.778	0.407
SPOT-RNA2	**0.676**	0.263	**0.709**	0.251	**0.781**	0.350
SPOT-RNA-2D-Single	0.556	0.523	0.560	0.545	0.680	0.717
SPOT-RNA-2D	0.668	**0.615**	0.658	**0.588**	0.711	**0.730**
Reduced test sets						
RNAContact	0.408	0.474	0.446	0.508	0.358	0.457
SPOT-RNA-2D-Single	0.532	0.491	0.620	0.546	0.674	0.711
SPOT-RNA-2D	**0.701**	**0.635**	**0.635**	**0.588**	**0.705**	**0.725**

*Note*: Comparison with SPOT-RNA and SPOT-RNA2 on reduced test sets TS1 (35 RNAs), TS2 (15 RNAs) and TS3 (54 RNAs) after removing the sequences overlapping with SPOT-RNA’s training data. Comparison with RNAContact on reduced test sets TS1 (21 RNAs), TS2 (9 RNAs) and TS3 (52 RNAs) after removing the sequences overlapping with RNAContact training data. Bold indicates the predictor with the best performance.

We also compared SPOT-RNA-2D and SPOT-RNA-2D-Single to our recently developed deep learning-based RNA secondary structure predictors SPOT-RNA and SPOT-RNA2. For a fair comparison, we removed test RNAs that have overlap with SPOT-RNA’s training data which reduced TS1 and TS2 to 38 and 16 RNAs, respectively. As shown in Table 3, SPOT-RNA-2D has a comparable mean F1-scores in base pairs and significantly better mean F1-score in non-base pairs when compared to deep-learning-based secondary-structure predictors. SPOT-RNA-2D improved long-range F1-score over RNAContact by 71%, 42% and 97% for base pairs and 34%, 16% and 59% for non-base pairs for test set TS1, TS2 and TS3, respectively. SPOT-RNA-2D-Single also outperforms RNAContact on three test sets (TS1, TS2 and TS3) without using any evolutionary features.

**Fig. 5.**
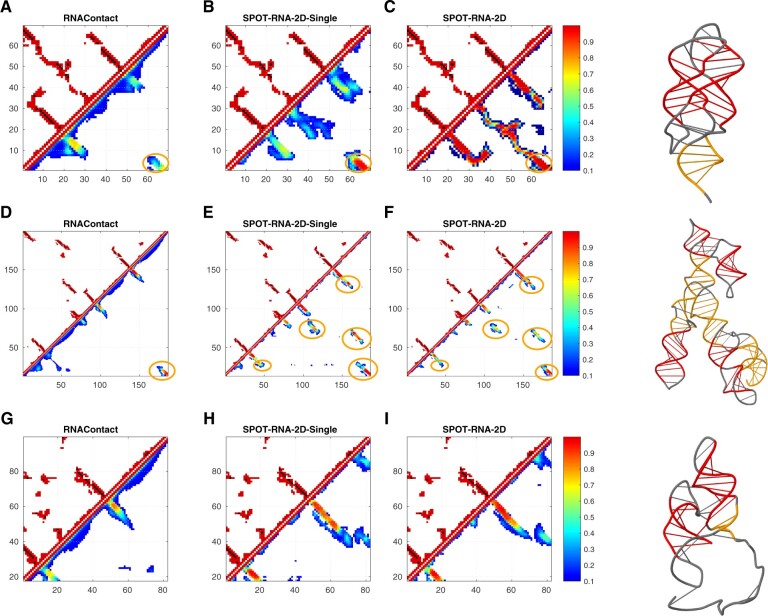
Comparison of predicted contact maps given by RNAContact (**A**, **D**, **G**), SPOT-RNA-2D-Single (**B**, **E**, **H**) and SPOT-RNA-2D (**C**, **F**, **I**) predicted contact map (in the lower triangle) with native contact map (in the upper triangle) for 2’-dG-II riboswitch (Chain A in PDB ID 6p2h), Varkud satellite ribozyme (Chain A in PDB ID 4r4v) and Hatchet Ribozyme (Chain A in PDB ID 6jq5) from RNA-Puzzles test set. Color bar indicates probability of predicted distance-based contact map in lower triangle. Highlighted orange circles indicate correctly predicted long-range contacts. Cartoon Figures indicate corresponding native 3D structure of upper triangular matrix on the left with long-range contacts highlighted by color orange and remaining contacts in color red (A color version of this figure appears in the online version of this article.)

Figure 5 illustrates predictions of RNAContact, SPOT-RNA-2D-Single and SPOT-RNA-2D predictions (in the lower triangle) and native contacts (in the upper triangle) for three RNAs from the RNA-Puzzles test set. Native contacts in the upper triangular matrix consist of both base pairs and non-base pairs. Base pairs are shown in the color black and non-base pairs in the color red. Also, long-range stems are highlighted in color orange in predicted contact maps and corresponding actual 3D structures, and remaining stems in color red in their corresponding actual 3D structures. Figure 5A–C shows the prediction of high *N*_eff_-value (*N*_eff_=94) *2’-dG-II riboswitch* (Matyjasik and Batey, 2019) (Chain A in PDB ID 6p2h, Puzzle-25) by RNAContact, SPOT-RNA-2D-Single and SPOT-RNA-2D, respectively. SPOT-RNA-2D is able to predict a native-contact like a pattern within top L long-range predicted contacts with a precision of 0.99. By comparison, SPOT-RNA-2D-Single and RNAContact can only achieve a precision of 0.64 and 0.59, respectively, whereas RNAContact missed the central helix region.

Figure 5D–F shows the prediction of *Varkud satellite ribozyme* (Suslov *et al.*, 2015) (Chain A in PDB ID 4r4v, Puzzle-7) with no evolutionary information (*N*_eff_=1) by RNAContact, SPOT-RNA-2D-Single and SPOT-RNA-2D, respectively. For this RNA, SPOT-RNA-2D-Single is the most accurate within top L long-range contacts with a precision of 0.83, followed by SPOT-RNA-2D (0.82) and RNAContact (0.42), respectively. This example illustrates SPOT-RNA-2D can essentially recover SPOT-RNA-2D-Single for low *N*_eff_ RNAs. RNAContact misses most structural features, whereas SPOT-RNA-2D captures most contact features.

Figure 5G–I shows the results of another low *N*_eff_ (*N*_eff_=3) RNA *hatchet ribozyme* (Zheng *et al.*, 2019) (Chain A in PDB ID 6jq5, Puzzle-22) by the RNAContact, SPOT-RNA-2D-Single and SPOT-RNA-2D, respectively. This RNA is relatively difficult to predict for all the predictors. The precision for top L long-range contacts was 0.35, 0.34 and 0.28 for SPOT-RNA-2D, SPOT-RNA-2D-Single and RNAContact, respectively. This RNA is more challenging because the contact map is dominated by many short-isolated contacts. These fragmented contacts were missed by all methods.

### 3.4 Tertiary structure modelling

Supplementary Table S5 summarizes the average RMSD of models from TS2 generated by Rosetta with the different input information. The baseline models [FARFAR2 (Watkins *et al.*, 2020)] rely only on a predicted secondary structure to guide minimization and yield an average RMSD of 16.9 Å for the top 1 low energy models. For TS2 it appears that there is not much advantage to using the true secondary structure labels compared with those predicted by RNAfold when few tertiary constraints are available (FARFAR2 and SPOT-RNA-2D-Single). Using the RNAfold predicted secondary structure combined with tertiary contacts predicted by SPOT-RNA-2D improves the mean RMSD to 14.6 Å (16% reduction). Results are much better if RNA complex structures are excluded (25 targets remained) with a 20% reduction from the mean RMSD of 15.5 Å by FARFAR2 to 12.7 Å by SPOT-RNA-2D. We also report the typical time taken to generate a single model by SPOT-RNA-2D compared with FARFAR2 in Supplementary Figure S5. Despite the computational overhead associated with evaluating tertiary constraints, the SPOT-RNA-2D computational time is comparable with FARFAR2 by forgoing the costly full atom refinement stage. Models were generated using a single core Intel(R) Xeon(R) CPU E5-2670 0 @ 2.60 GHz. Models generated with DSSR secondary structure and constraints derived from true contacts in the native structure represent a lower bound on modelling performance within the proposed framework. However, even with perfect contact prediction and a perfect secondary structure, only a mean RMSD of 8.1 Å (6.5 Å excluding complex structures) can be achieved. This suggests that even using perfect contact information is not sufficient to fold with Rosetta.

An example that demonstrates the efficacy of the SPOT-RNA-2D predicted contacts is the 2’-dG riboswitch (Pikovskaya *et al.*, 2011) (Supplementary Fig. S6). Black dots in the upper diagonal of the contact maps represent base-paired nucleotides predicted by RNAfold and derived from DSSR in the native structure. The black color in the lower diagonal represents constraints that are satisfied by the top 1 model and the blue color is used to indicate constraints that were not satisfied by the respective model. While the secondary structure is correctly assigned by RNAfold, relative helix orientations are not recovered by the naïve FARFAR2 protocol which leads to a poor RMSD of the top 1 model with the native structure (17.7 Å). The SPOT-RNA-2D predicted contact map includes critical tertiary interactions which enforce the correct antiparallel helical configuration when applied as constraints and significantly improve the RMSD to 6.6 Å. The evolution-derived, residue constraints bias the conformation sampling toward the native structure leading to a characteristic ‘folding funnel’ which is not observed when ignoring tertiary constraints or when using those derived from SPOT-RNA-2D and SPOT-RNA-2D-Single (Supplementary Fig. S7).

## 4 Discussion

Initial advances in protein structure prediction using deep learning [CASP11 (Monastyrskyy *et al.*, 2016) and CASP12 (Schaarschmidt *et al.*, 2018)] can be largely attributed to the large improvement in protein contact-map prediction. Thus, it is critical to improve the prediction of RNA contact map for advancing RNA structure prediction. Most previous work on RNA base-base contacts was limited to base pairs in terms of secondary structure prediction, evolutionary coupling analysis or deep learning on the evolutionary-derived 1D profile. Here, we developed SPOT-RNA-2D that provides integrated deep learning on physics-derived base pairs and evolutionary-derived direct coupling for improving distance-based contact prediction. The new method substantially improves over existing techniques across different test sets regardless of the structures were determined by X-ray crystallography or NMR techniques.

One nice feature of SPOT-RNA-2D is that it can recover the performance of the single-sequence-based method SPOT-RNA-2D-Single when few homologous sequences are available. This is supported by the dependence of the performance of SPOT-RNA-2D and SPOT-RNA-2D-Single on the number of effective homologous sequences (Fig. 4). Thus, SPOT-RNA-2D is able to better generalize performance across different *N*_eff_-values due to MSA sampling used during training. By comparison, the performance of RNAContact does not have a strong dependence on *N*_eff_, suggesting that a 1D PSSM profile based on sequence-derived homolog is insufficient to capture the full extent of evolution information. More importantly, when *N*_eff_>50, SPOT-RNA-2D can make highly accurate performance (80% precision for top L long-range contact prediction).

One limitation of SPOT-RNA-2D is due to its usage of RNAcmap to derive evolution information. RNAcmap uses BLAST-N to make the first round of sequence-based search for homologs, followed by INFERNAL to perform the second round of sequence-profile and secondary-structure-based search. This method is computationally prohibitive for RNA sequences >1000 bases. As a result, our model was trained with a maximum sequence length of 418. For example, RNAcamp can take 5–6 h on 40 CPU threads for 418 bases RNA sequence, which will be the same for all the alignment-based predictors. After that, SPOT-RNA-2D only takes 92.68 s for making the prediction of all RNAs in the test set TS1. For time-sensitive calculations, SPOT-RNA-2D-Single can be used, which takes only 43.76 s for full test set TS1 prediction from single-sequence and performs better than the RNAContact and DCA predictors, and comparable to SPOT-RNA-2D on low *N*_eff_ RNAs.

One disappointing result is that even with perfect secondary-structure base-pair assignment and tertiary contact accuracy, the average RMSD is still 8.1 Å for targets in TS2 (6.5 Å after excluding complex structures). This highlights a potential limitation of the current modelling framework. One likely reason is the lack of backbone restraints, which has been proven important as secondary structure restraints in protein structure prediction. Our recent result shows that backbone angles can be predicted with reasonable accuracy (Singh *et al.*, 2021b), suggesting the possibility of using both predicted backbone angles and contact maps as restraints for RNA structure prediction. Another possibility is to make an end-to-end the prediction like AlphaFold2 (Jumper *et al.*, 2021) and RoseTTAfold (Baek *et al.*, 2021). However, it is not clear if there are sufficient structural data to make an accurate inference.

To place the accuracy for RNA contact prediction in context, we compare it to the accuracy of protein contact prediction. The average number of RNA contacts is about 3 L (Supplementary Table S1), compared to 1.37 L for 669 proteins in SPOT-2018 (Singh *et al.*, 2021c) set based on an 8 Å cutoff. That is, RNAs have about twice contacts than proteins for the same cutoff. In other words, top L/2 contacts of proteins would cover a similar fraction of native contacts for top L contacts of RNAs. According to recent work in protein contact predictions trRosetta, the precision for top L/2 long-range contacts is 54–67% for CASP-13 FM targets by RaptorX-Contact (Wang *et al.*, 2017b), TripletRes (Li *et al.*, 2021), trRosetta (Yang *et al.*, 2020) and 66–72% on the CAMEO set by trRosetta models. By comparison, the precision for top L long-range contacts for RNAs given by SPOT-RNA-2D is 66–74% for three test sets (TS1, TS2, TS3). Thus, the overall accuracy for RNA contact prediction has reached the same level as that for protein contact prediction. In other words, the time has come to use predicted RNA contacts as restraints for RNA structure prediction.

## Supplementary Material

btac421_Supplementary_Data

## References

[btac421-B1] Altschul S.F. et al (1997) Gapped BLAST and PSI-BLAST: a new generation of protein database search programs. Nucleic Acids Res., 25, 3389–3402. 9254694 10.1093/nar/25.17.3389 PMC146917

[btac421-B2] Ba J.L. et al (2016) Layer normalization. *Preprint* arXiv, 1*607.06450.*

[btac421-B3] Baek M. et al (2021) Accurate prediction of protein structures and interactions using a three-track neural network. Science, 373, 871–876. 34282049 10.1126/science.abj8754 PMC7612213

[btac421-B4] Balakrishnan S. et al (2011) Learning generative models for protein fold families. Proteins Struct. Funct. Bioinform., 79, 1061–1078. 10.1002/prot.22934 21268112

[btac421-B5] Cai Z. et al (2020) RIC-seq for global in situ profiling of RNA–RNA spatial interactions. Nature, 582, 432–437. 32499643 10.1038/s41586-020-2249-1

[btac421-B6] Carlson P.D. et al (2018) SnapShot: RNA structure probing technologies. Cell, 175, 600–600.e1. 30290145 10.1016/j.cell.2018.09.024 PMC6582637

[btac421-B7] Clevert D.-A. et al (2015) Fast and accurate deep network learning by exponential linear units (ELUs). *Preprint* arXiv, *1511.07289*

[btac421-B8] Cock P.J.A. et al (2009) Biopython: freely available Python tools for computational molecular biology and bioinformatics. Bioinformatics, 25, 1422–1423. 19304878 10.1093/bioinformatics/btp163 PMC2682512

[btac421-B9] Consortium, R. (2020) RNAcentral 2021: secondary structure integration, improved sequence search and new member databases. Nucleic Acids Res., 49, D212–D220. 10.1093/nar/gkaa921 PMC7779037 33106848

[btac421-B10] Cruz J.A. et al (2012) RNA-Puzzles: a CASP-like evaluation of RNA three-dimensional structure prediction. RNA, 18, 610–625. 22361291 10.1261/rna.031054.111 PMC3312550

[btac421-B11] De Leonardis E. et al (2015a) Direct-coupling analysis of nucleotide coevolution facilitates RNA secondary and tertiary structure prediction. Nucleic Acids Res., 43, 10444–10455. 26420827 10.1093/nar/gkv932 PMC4666395

[btac421-B12] De Leonardis E. et al (2015b) Direct-coupling analysis of nucleotide coevolution facilitates RNA secondary and tertiary structure prediction. Nucleic Acids Res., 43, 10444–10455. 26420827 10.1093/nar/gkv932 PMC4666395

[btac421-B13] Ekeberg M. et al (2013) Improved contact prediction in proteins: using pseudolikelihoods to infer Potts models. Phys. Rev. E Stat. Nonlin. Soft Matter Phys., 87, 012707. 23410359 10.1103/PhysRevE.87.012707

[btac421-B14] Fu L. et al (2012) CD-HIT: accelerated for clustering the next-generation sequencing data. Bioinformatics, 28, 3150–3152. 23060610 10.1093/bioinformatics/bts565 PMC3516142

[btac421-B15] Hanson J. et al (2018) Accurate prediction of protein contact maps by coupling residual two-dimensional bidirectional long short-term memory with convolutional neural networks. Bioinformatics, 34, 4039–4045. 29931279 10.1093/bioinformatics/bty481

[btac421-B16] Hanumanthappa A.K. et al (2021) Single-sequence and profile-based prediction of RNA solvent accessibility using dilated convolutional neural network. Bioinformatics, 36, 5169–5176. 33106872 10.1093/bioinformatics/btaa652

[btac421-B17] He K. et al (2016) Identity mappings in deep residual networks. In: Leibe B. et al (eds.) Computer Vision – ECCV 2016. Springer International Publishing, Cham, pp. 630–645.

[btac421-B18] Hopf T.A. et al (2017) Mutation effects predicted from sequence co-variation. Nat. Biotechnol., 35, 128–135. 28092658 10.1038/nbt.3769 PMC5383098

[btac421-B19] Janssen S. , Giegerich R. (2015) The RNA shapes studio. Bioinformatics, 31, 423–425. 25273103 10.1093/bioinformatics/btu649 PMC4308662

[btac421-B20] Jian Y. et al (2019) DIRECT: RNA contact predictions by integrating structural patterns. BMC Bioinformatics, 20, 497. 31615418 10.1186/s12859-019-3099-4 PMC6794908

[btac421-B21] Jumper J. et al (2021) Highly accurate protein structure prediction with AlphaFold. Nature, 596, 583–589. 34265844 10.1038/s41586-021-03819-2 PMC8371605

[btac421-B22] Kalvari I. et al (2018) Rfam 13.0: shifting to a genome-centric resource for non-coding RNA families. Nucleic Acids Res., 46, D335–D342. 29112718 10.1093/nar/gkx1038 PMC5753348

[btac421-B23] Kamisetty H. et al (2013) Assessing the utility of coevolution-based residue–residue contact predictions in a sequence- and structure-rich era. Proc. Natl. Acad. Sci. USA, 110, 15674–15679. 24009338 10.1073/pnas.1314045110 PMC3785744

[btac421-B24] Kingma D.P. , Ba J. (2014) Adam: a method for stochastic optimization. *Preprint arXiv*, *1511.07122*

[btac421-B25] Kubota M. et al (2015) Progress and challenges for chemical probing of RNA structure inside living cells. Nat. Chem. Biol., 11, 933–941. 26575240 10.1038/nchembio.1958 PMC5068366

[btac421-B26] Li Y. et al (2021) Deducing high-accuracy protein contact-maps from a triplet of coevolutionary matrices through deep residual convolutional networks. PLoS Comput. Biol., 17, e1008865. 33770072 10.1371/journal.pcbi.1008865 PMC8026059

[btac421-B27] Lorenz R. et al (2011) ViennaRNA package 2.0. Algorithms Mol. Biol., 6, 26. 22115189 10.1186/1748-7188-6-26 PMC3319429

[btac421-B28] Lu X.-J. et al (2015) DSSR: an integrated software tool for dissecting the spatial structure of RNA. Nucleic Acids Res., 43, e142. 26184874 10.1093/nar/gkv716 PMC4666379

[btac421-B29] Luo Q.-J. et al (2021) RNA structure probing reveals the structural basis of Dicer binding and cleavage. Nat. Commun., 12, 3397. 34099665 10.1038/s41467-021-23607-w PMC8184798

[btac421-B30] Matyjasik M.M. , Batey R.T. (2019) Structural basis for 2’-deoxyguanosine recognition by the 2’-dG-II class of riboswitches. Nucleic Acids Res., 47, 10931–10941. 31598729 10.1093/nar/gkz839 PMC6847200

[btac421-B31] Miao Z. et al (2015) RNA-puzzles round II: assessment of RNA structure prediction programs applied to three large RNA structures. RNA, 21, 1066–1084. 25883046 10.1261/rna.049502.114 PMC4436661

[btac421-B32] Miao Z. et al (2017) RNA-puzzles round III: 3D RNA structure prediction of five riboswitches and one ribozyme. RNA, 23, 655–672. 28138060 10.1261/rna.060368.116 PMC5393176

[btac421-B33] Miao Z. et al (2020) RNA-puzzles round IV: 3D structure predictions of four ribozymes and two aptamers. RNA (New York, N.Y.), 26, 982–995. 32371455 10.1261/rna.075341.120 PMC7373991

[btac421-B34] Monastyrskyy B. et al (2016) New encouraging developments in contact prediction: assessment of the CASP11 results. Proteins, 84, 131–144. 26474083 10.1002/prot.24943 PMC4834069

[btac421-B35] Morcos F. et al (2011) Direct-coupling analysis of residue coevolution captures native contacts across many protein families. Proc. Natl. Acad. Sci. USA, 108, E1293–E1301. 22106262 10.1073/pnas.1111471108 PMC3241805

[btac421-B36] Nawrocki E.P. , Eddy S.R. (2013) Infernal 1.1: 100-fold faster RNA homology searches. Bioinformatics, 29, 2933–2935. 24008419 10.1093/bioinformatics/btt509 PMC3810854

[btac421-B37] Pikovskaya O. et al (2011) Structural principles of nucleoside selectivity in a 2’-deoxyguanosine riboswitch. Nat. Chem. Biol., 7, 748–755. 21841796 10.1038/nchembio.631 PMC3781940

[btac421-B38] Pucci F. et al (2020) Evaluating DCA-based method performances for RNA contact prediction by a well-curated data set. RNA, 26, 794–802. 32276988 10.1261/rna.073809.119 PMC7297115

[btac421-B39] Reuter J.S. , Mathews D.H. (2010) RNAstructure: software for RNA secondary structure prediction and analysis. BMC Bioinformatics, 11, 129. 20230624 10.1186/1471-2105-11-129 PMC2984261

[btac421-B40] Rose P.W. et al (2017) The RCSB protein data bank: integrative view of protein, gene and 3D structural information. Nucleic Acids Res., 45, D271–D281. 27794042 10.1093/nar/gkw1000 PMC5210513

[btac421-B41] Schaarschmidt J. et al (2018) Assessment of contact predictions in casp12: co-evolution and deep learning coming of age. Proteins, 86, 51–66. 29071738 10.1002/prot.25407 PMC5820169

[btac421-B42] Schug A. et al (2009) High-resolution protein complexes from integrating genomic information with molecular simulation. Proc. Natl. Acad. Sci. USA, 106, 22124–22129. 20018738 10.1073/pnas.0912100106 PMC2799721

[btac421-B43] Senior A.W. et al (2020) Improved protein structure prediction using potentials from deep learning. Nature, 577, 706–710. 31942072 10.1038/s41586-019-1923-7

[btac421-B44] Singh J. et al (2019) RNA secondary structure prediction using an ensemble of two-dimensional deep neural networks and transfer learning. Nat. Commun., 10, 5407. 31776342 10.1038/s41467-019-13395-9 PMC6881452

[btac421-B45] Singh J. et al (2021a) Improved RNA secondary structure and tertiary base-pairing prediction using evolutionary profile, mutational coupling and two-dimensional transfer learning. Bioinformatics, 37, 2589–2600. 33704363 10.1093/bioinformatics/btab165

[btac421-B46] Singh J. et al (2021b) RNA backbone torsion and pseudotorsion angle prediction using dilated convolutional neural networks. J. Chem. Inf. Model., 61, 2610–2622. 34037398 10.1021/acs.jcim.1c00153

[btac421-B47] Singh J. et al (2021c) SPOT-1D-Single: improving the single-sequence-based prediction of protein secondary structure, backbone angles, solvent accessibility and half-sphere exposures using a large training set and ensembled deep learning. Bioinformatics, 37, 3464–3472. 33983382 10.1093/bioinformatics/btab316

[btac421-B48] Solayman M. et al (2022) Probing RNA structures and functions by solvent accessibility: an overview from experimental and computational perspectives. Briefings in Bioinformatics, 23, bbac112. 35348613 10.1093/bib/bbac112 PMC9116373

[btac421-B49] Sun S. et al (2019) Enhanced prediction of RNA solvent accessibility with long short-term memory neural networks and improved sequence profiles. Bioinformatics, 35, 1686–1691. 30321300 10.1093/bioinformatics/bty876

[btac421-B50] Sun S. et al (2021) RNA inter-nucleotide 3D closeness prediction by deep residual neural networks. Bioinformatics, 37, 1093–1098. 33135062 10.1093/bioinformatics/btaa932 PMC8150135

[btac421-B51] Suslov N.B. et al (2015) Crystal structure of the Varkud satellite ribozyme. Nat. Chem. Biol., 11, 840–846. 26414446 10.1038/nchembio.1929 PMC4618023

[btac421-B52] Tinoco I. , Bustamante C. (1999) How RNA folds. J. Mol. Biol., 293, 271–281. 10550208 10.1006/jmbi.1999.3001

[btac421-B53] Wang J. et al (2017a) Optimization of RNA 3D structure prediction using evolutionary restraints of nucleotide-nucleotide interactions from direct coupling analysis. Nucleic Acids Res., 45, 6299–6309. 28482022 10.1093/nar/gkx386 PMC5499770

[btac421-B54] Wang S. et al (2017b) Accurate de novo prediction of protein contact map by ultra-deep learning model. PLoS Comput. Biol., 13, e1005324. 28056090 10.1371/journal.pcbi.1005324 PMC5249242

[btac421-B55] Watkins A.M. et al (2020) FARFAR2: improved De novo rosetta prediction of complex global RNA folds. Structure, 28, 963–976.e6. 32531203 10.1016/j.str.2020.05.011 PMC7415647

[btac421-B56] Weigt M. et al (2009) Identification of direct residue contacts in protein–protein interaction by message passing. Proc. Natl. Acad. Sci. USA, 106, 67–72. 19116270 10.1073/pnas.0805923106 PMC2629192

[btac421-B57] Weinreb C. et al (2016) 3D RNA and functional interactions from evolutionary couplings. Cell, 165, 963–975. 27087444 10.1016/j.cell.2016.03.030 PMC5024353

[btac421-B58] Yang J. et al (2020) Improved protein structure prediction using predicted interresidue orientations. Proc. Natl. Acad. Sci. USA, 117, 1496–1503. 31896580 10.1073/pnas.1914677117 PMC6983395

[btac421-B59] Zerihun M.B. et al (2020) pydca v1.0: a comprehensive software for direct coupling analysis of RNA and protein sequences. Bioinformatics, 36, 2264–2265. 31778142 10.1093/bioinformatics/btz892

[btac421-B60] Zhang H. et al (2020a) LinearPartition: linear-time approximation of RNA folding partition function and base-pairing probabilities. Bioinformatics, 36, i258–i267. 32657379 10.1093/bioinformatics/btaa460 PMC7355276

[btac421-B61] Zhang Y. et al (2020b) 3dRNA: building RNA 3D structure with improved template library. Comput. Struct. Biotechnol. J., 18, 2416–2423. 33005304 10.1016/j.csbj.2020.08.017 PMC7508704

[btac421-B62] Zhang Z. et al (2020c) Accurate inference of the full base-pairing structure of RNA by deep mutational scanning and covariation-induced deviation of activity. Nucleic Acids Res., 48, 1451–1465. 31872260 10.1093/nar/gkz1192 PMC7026644

[btac421-B63] Zhang T. et al (2021) RNAcmap: a fully automatic pipeline for predicting contact maps of RNAs by evolutionary coupling analysis. Bioinformatics, 37, 3494–3500. 34021744 10.1093/bioinformatics/btab391

[btac421-B64] Zheng L. et al (2019) Hatchet ribozyme structure and implications for cleavage mechanism. Proc. Natl. Acad. Sci. USA, 116, 10783–10791. 31088965 10.1073/pnas.1902413116 PMC6561176

