# Supplementary material for: Predicting RNA distance-based contact maps by integrated deep learning on physics-inferred secondary structure and evolutionary-derived mutational coupling
Source: Bioinformatics. 2022 Jun 25;38(16):3900–10. doi: 10.1093/bioinformatics/btac421 (PMC9364379; doi:10.1093/bioinformatics/btac421)
Supplement: btac421_Supplementary_Data [file btac421_supplementary_data.pdf]

Supplementary Material: Predicting RNA distance-based contact maps by integrated deep learning on physics-inferred secondary structure and evolutionary-derived mutational coupling

Jaswinder Singh[1,*], Kuldip Paliwal[1,*], Thomas Litfin[2], Jaspreet Singh[1], and Yaoqi Zhou[2,3,4,*]

[1]Signal Processing Laboratory, School of Engineering and Built Environment, Griffith University, Brisbane, QLD 4111, Australia
[2]Institute for Glycomics, Griffith University, Parklands Dr. Southport, QLD 4222, Australia
[3]Institute for Systems and Physical Biology, Shenzhen Bay Laboratory, Shenzhen 518055, China.
[4]Peking University Shenzhen Graduate School, Shenzhen, 518055, P.R. China.
[*]Correspondence to yaoqi.zhou@griffith.edu.au, jaswinder.singh3@griffithuni.edu.au, and k.paliwal@griffith.edu.au

Table S1: The number of RNAs, the number of various base pairs, and the sequence lengths of the training (TR), validation (VL), and three independent test sets (TS1, TS2, and TS3).
(Note: The number inside the parentheses indicates the percentage of total base pairs.)

	No. of RNAs	Canonical Base-Pairs		Noncanonical Base-Pairs	Multiplets Base-Pairs	Distance-based contacts statistics		Median sequence length	Maximum sequence length	Median N_{eff}-Value
		Watson-Crick	Wobble			Average Contacts	Standard deviation			
TR	286	7761 (68.7%)	930 (8.2%)	2600 (23.0%)	3380 (29.9%)	3.17	0.72	76.0	418	188.42
VL	30	372 (69.0%)	39 (7.2%)	128 (23.7%)	164 (30.4%)	2.48	0.85	40.5	64	2.00
TS1	63	1452 (69.2%)	168 (8.0%)	477 (22.7%)	553 (26.4%)	2.96	0.70	75.0	186	40.16
TS2	30	720 (71.5%)	84 (8.3%)	203 (20.2%)	256 (25.4%)	3.07	0.71	68.0	174	9.13
TS3	54	822 (73.9%)	102 (9.2%)	189 (17.0%)	237 (21.3%)	2.53	0.49	40.5	103	3.77

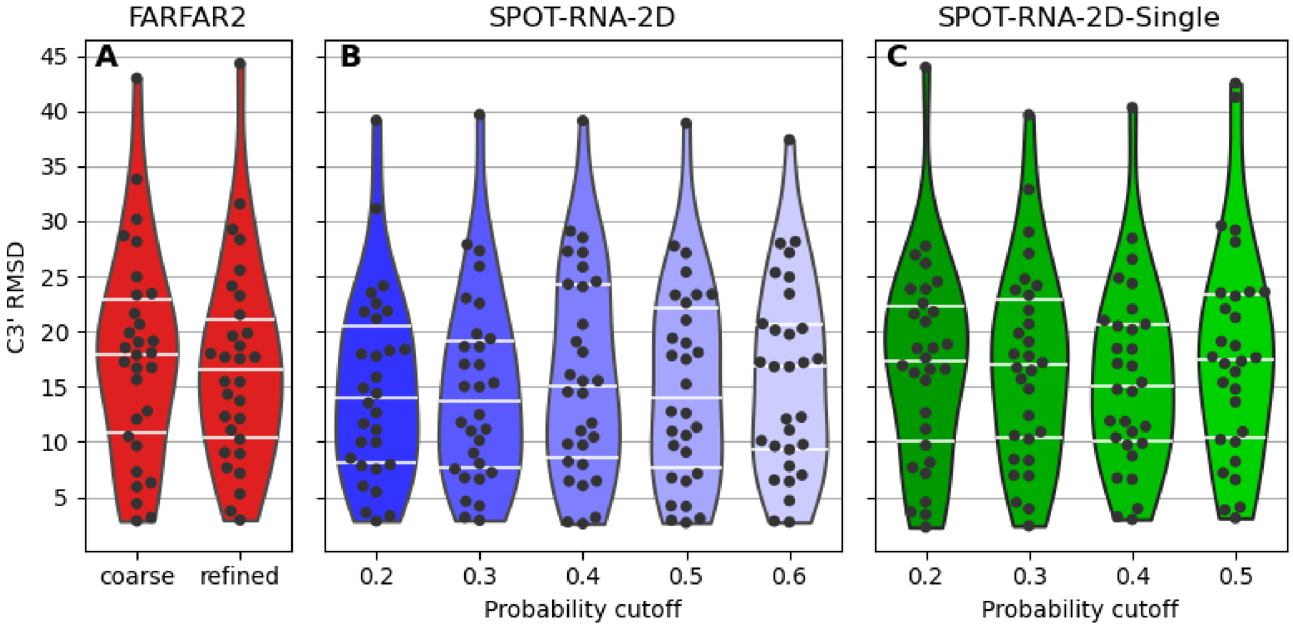

Figure S1. Distribution of C3' RMSD between top 1 low energy model and native structures for TS2 dataset used to optimize conditions for (A) FARFAR2, (B) SPOT-RNA-2D and (C) SPOT-RNA-2D-Single

Table S2: The number of RNAs in Rfam families from training (TR), validation (VL), and two independent test sets (TS1 and TS2).

	Number of RNAs			
Rfam family	Train set (TR)	Validation set (VL)	Test set (TS1)	Test set (TS2)
RF00005	80	-	14	-
RF00162	22	-	1	-
RF00167	22	-	1	-
RF00379	12	-	-	-
RF02001	9	-	-	-
RF00001	6	-	2	-
RF00234	5	-	-	-
RF02541	5	-	1	-
RF01051	5	-	1	-
RF02679	3	-	-	-
RF00059	3	-	1	-
RF00017	3	-	-	-
RF01852	3	-	1	-
RF01831	2	-	1	-
RF01854	2	-	-	-
RF00026	2	-	-	-
RF02553	1	-	-	-
RF02540	1	-	-	-
RF01826	1	-	-	-
RF00458	1	-	-	-
RF00008	1	-	-	1
RF00010	1	-	-	-
RF00390	1	-	-	-
RF00050	1	-	-	-
RF00233	1	-	-	-
RF00442	1	-	-	-
RF00505	1	-	-	-
RF02683	1	-	-	-
RF02680	1	-	-	-
RF01344	1	-	-	-
RF01704	-	1	-	-
RF03013	-	1	-	-
RF01734	-	1	-	-
RF02519	-	1	-	-
RF01763	-	1	-	-
RF02796	-	1	-	-
RF01750	-	-	2	-
RF00102	-	-	1	-
RF01725	-	-	1	-
RF01857	-	-	1	-
RF04190	-	-	1	-
RF00029	-	-	1	-
RF01415	-	-	1	-
RF00168	-	-	-	1
RF00228	-	-	-	1
RF00380	-	-	-	1
RF00002	-	-	-	1
RF00174	-	-	-	1
RF00164	-	-	-	1
RF01786	-	-	-	1
RF00504	-	-	-	1
RF00100	-	-	-	1
RF02678	-	-	-	1
RF01689	-	-	-	1
No Rfam family	88	24	32	18

Table S3. SPOT-RNA2 ensemble architectures.

	Number of Blocks-A (N_A)	Number of filters (N_F)
Model-0	20	40
Model-1	6	56
Model-2	10	64
Model-3	16	72

Table S4: Performance of SPOT-RNA-2D on individual Rfam families on 93 RNAs from two independent test sets (TS1 and TS2). Rfam families highlighted in color red exists in training data.

Rfam family (No. of RNAs)	MCC	F1	Precision	Sensitivity
RF00001 (2)	0.590	0.612	0.748	0.518
RF00002 (1)	0.521	0.545	0.585	0.509
RF00005 (14)	0.648	0.681	0.812	0.586
RF00008 (1)	0.759	0.776	0.949	0.656
RF00029 (1)	0.849	0.865	0.936	0.804
RF00059 (1)	0.585	0.608	0.841	0.476
RF00100 (1)	0.754	0.778	0.951	0.658
RF00102 (1)	0.762	0.766	0.957	0.639
RF00162 (1)	0.747	0.756	0.908	0.647
RF00164 (1)	0.740	0.770	0.983	0.633
RF00167 (1)	0.673	0.676	0.978	0.517
RF00168 (1)	0.744	0.736	0.960	0.597
RF00174 (1)	0.702	0.706	0.875	0.592
RF00228 (1)	0.795	0.808	0.912	0.726
RF00380 (1)	0.645	0.644	0.871	0.510
RF00504 (1)	0.757	0.769	0.945	0.648
RF01051 (1)	0.612	0.630	0.849	0.501
RF01415 (1)	0.532	0.573	0.739	0.468
RF01689 (1)	0.586	0.607	0.786	0.494
RF01725 (1)	0.726	0.737	0.917	0.617
RF01750 (2)	0.730	0.747	0.930	0.624
RF01786 (1)	0.610	0.638	0.820	0.523
RF01831 (1)	0.729	0.748	0.848	0.669
RF01852 (1)	0.802	0.817	0.922	0.733
RF01857 (1)	0.806	0.809	0.958	0.700
RF02541 (1)	0.668	0.663	0.980	0.501
RF02678 (1)	0.722	0.734	0.920	0.611
RF04190 (1)	0.827	0.837	0.938	0.755
No Rfam family (50)	0.749	0.769	0.896	0.674

Table S5. Performance comparison in terms of mean C3' RMSD for 30 targets from TS2 generated without tertiary constraints (FARFAR2), with predicted tertiary constraints by SPOT-RNA-2D-Single and SPOT-RNA-2D and with tertiary constraints derived from the native structure (Native). Bold indicates the predictor with the best performance. RNAfold (SS) means predicted secondary structure from RNAfold and DSSR (SS) means secondary structure derived from native 3D structure using DSSR tool.

	RNAfold (SS)	DSSR (SS)
FARFAR2	16.9	17.7
SPOT-RNA-2D-Single	15.7	16.1
SPOT-RNA-2D	14.6	13.8
Native	**10.3**	**8.1**

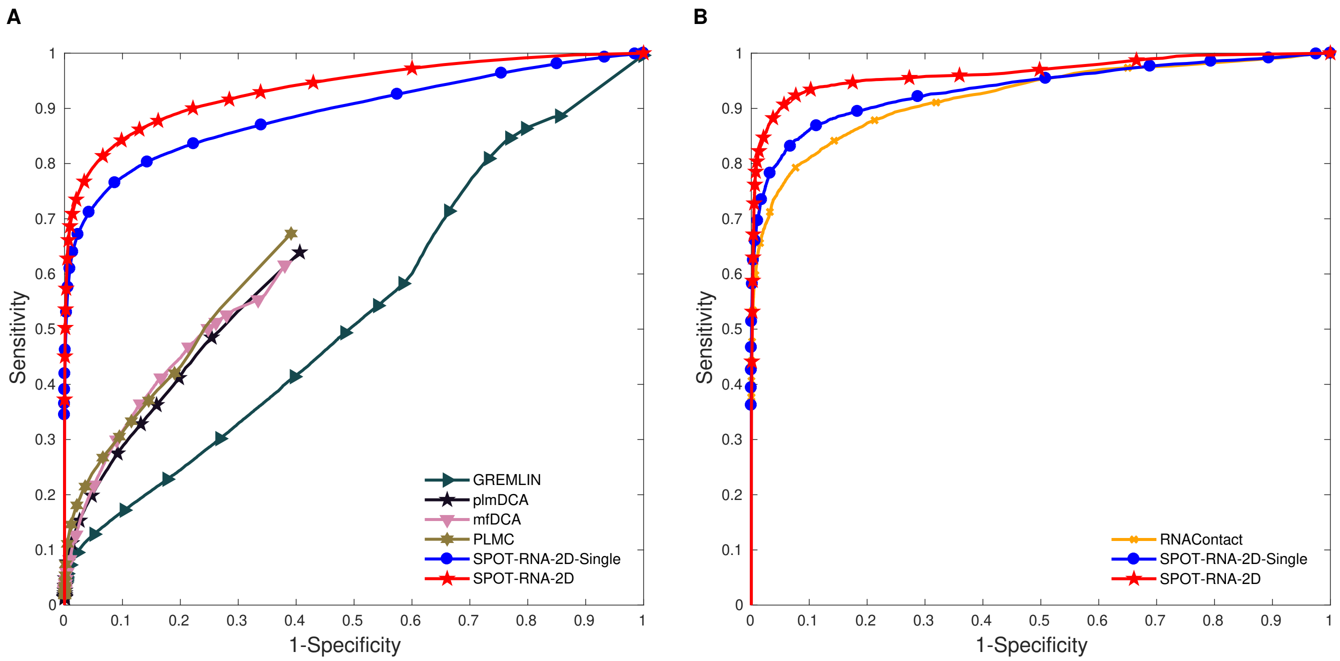

Figure S2. Receiver Operating Curves (ROC) curves given by SPOT-RNA-2D and SPOT-RNA-2D-Single **(A)** along with four DCA predictors on 147 RNAs from three test sets TS1, TS2, and TS3, **(B)** Further comparison with RNAContact on 82 RNAs from three reduced test sets TS1, TS2, and TS3 after removing the sequences overlapping with RNAContact training data.

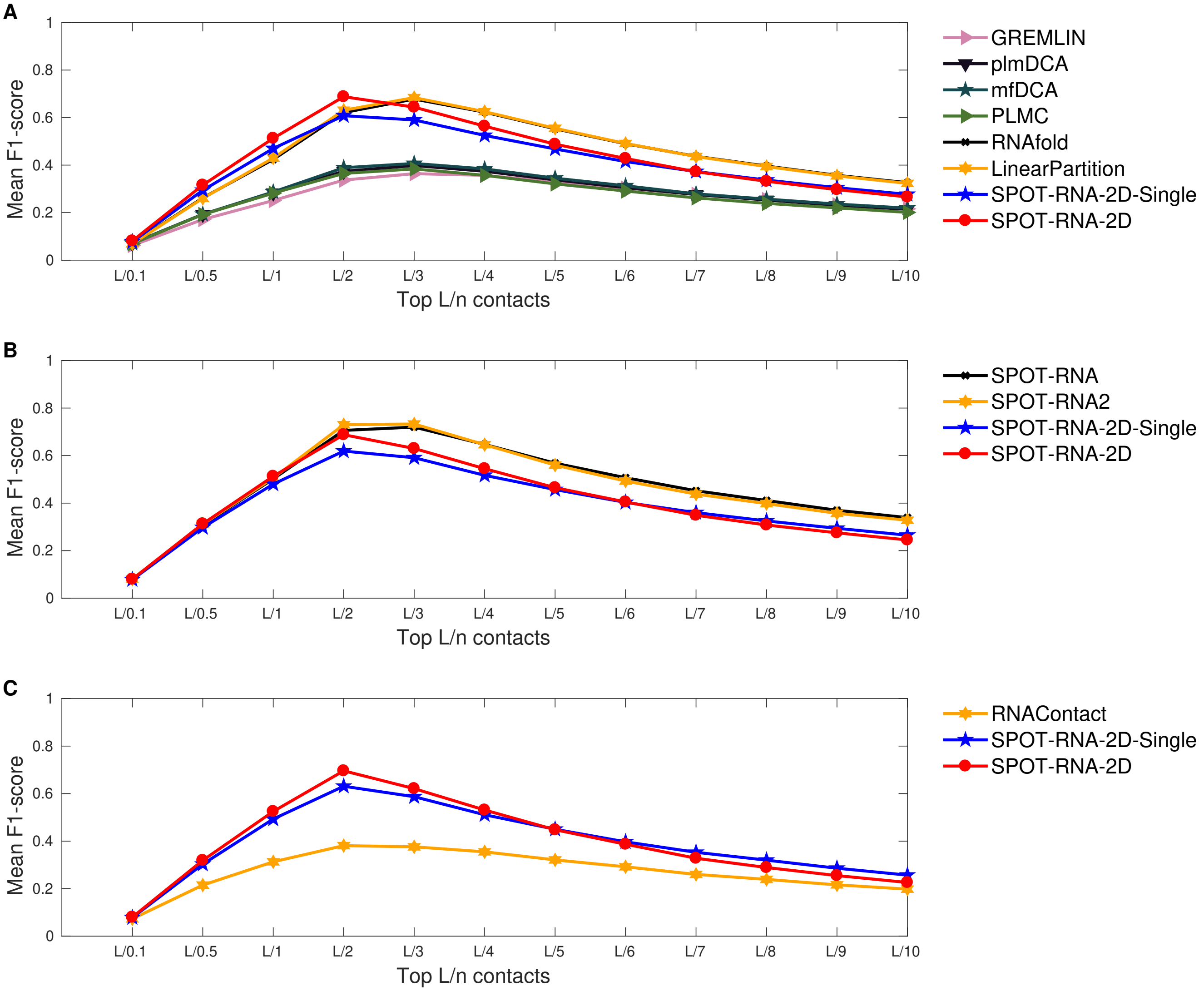

Figure S3. Mean F1-score as a function of top L/n base pairs given by **(A)** SPOT-RNA-2D, SPOT-RNA-2D-Single, four DCA predictors (GREMLIN, plmDCA, mfDCA, and PLMC), and two secondary structure predictors (RNAfold and LinearPartition) on combined full test sets (TS1, TS2, and TS3), **(B)** SPOT-RNA-2D, SPOT-RNA-2D-Single, and two deep learning-based secondary structure predictors (SPOT-RNA and SPOT-RNA2) on combined reduced test sets TS1 (35 RNAs), TS2 (15 RNAs) and TS3 (54 RNAs) after removing the sequences overlapping with SPOT-RNA's training data, **(C)** SPOT-RNA-2D, SPOT-RNA-2D-Single and RNAContact on combined reduced test sets TS1 (21 RNAs), TS2 (9 RNAs) and TS3 (52 RNAs) after removing the sequences overlapping with RNAContact training data.

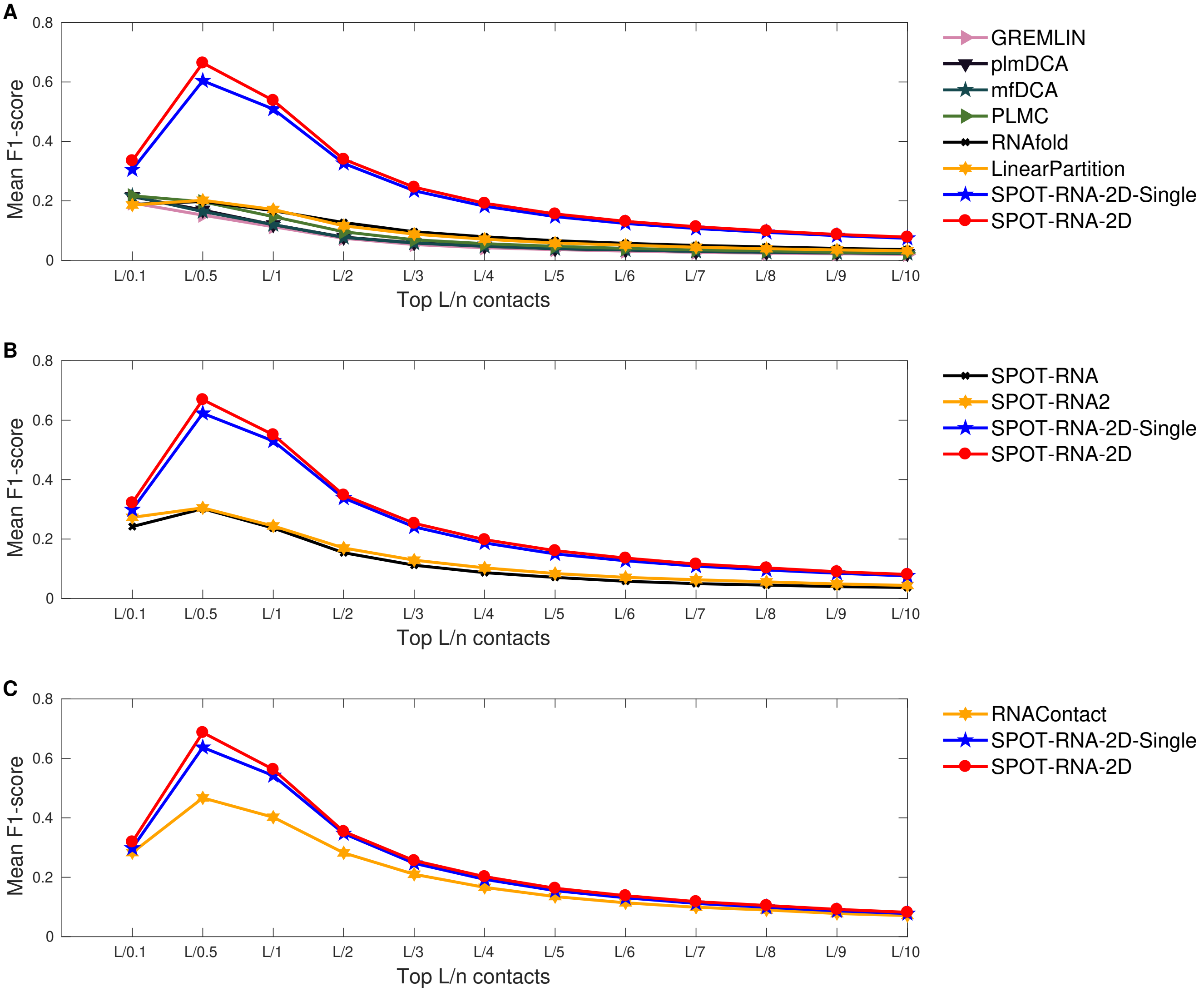

Figure S4. Mean F1-score as a function of top L/n non-local $[(i-j) \geq 6]$ non-base pairs given by **(A)** SPOT-RNA-2D, SPOT-RNA-2D-Single, four DCA predictors (GREMLIN, plmDCA, mfDCA, and PLMC), and two secondary structure predictors (RNAfold and LinearPartition) on combined full test sets (TS1, TS2, and TS3), **(B)** SPOT-RNA-2D, SPOT-RNA-2D-Single, and two deep learning-based secondary structure predictors (SPOT-RNA and SPOT-RNA2) on combined reduced test sets TS1 (35 RNAs), TS2 (15 RNAs) and TS3 (54 RNAs) after removing the sequences overlapping with SPOT-RNA's training data, **(C)** SPOT-RNA-2D, SPOT-RNA-2D-Single, and RNAContact on combined reduced test sets TS1 (21 RNAs), TS2 (9 RNAs) and TS3 (52 RNAs) after removing the sequences overlapping with RNAContact training data..

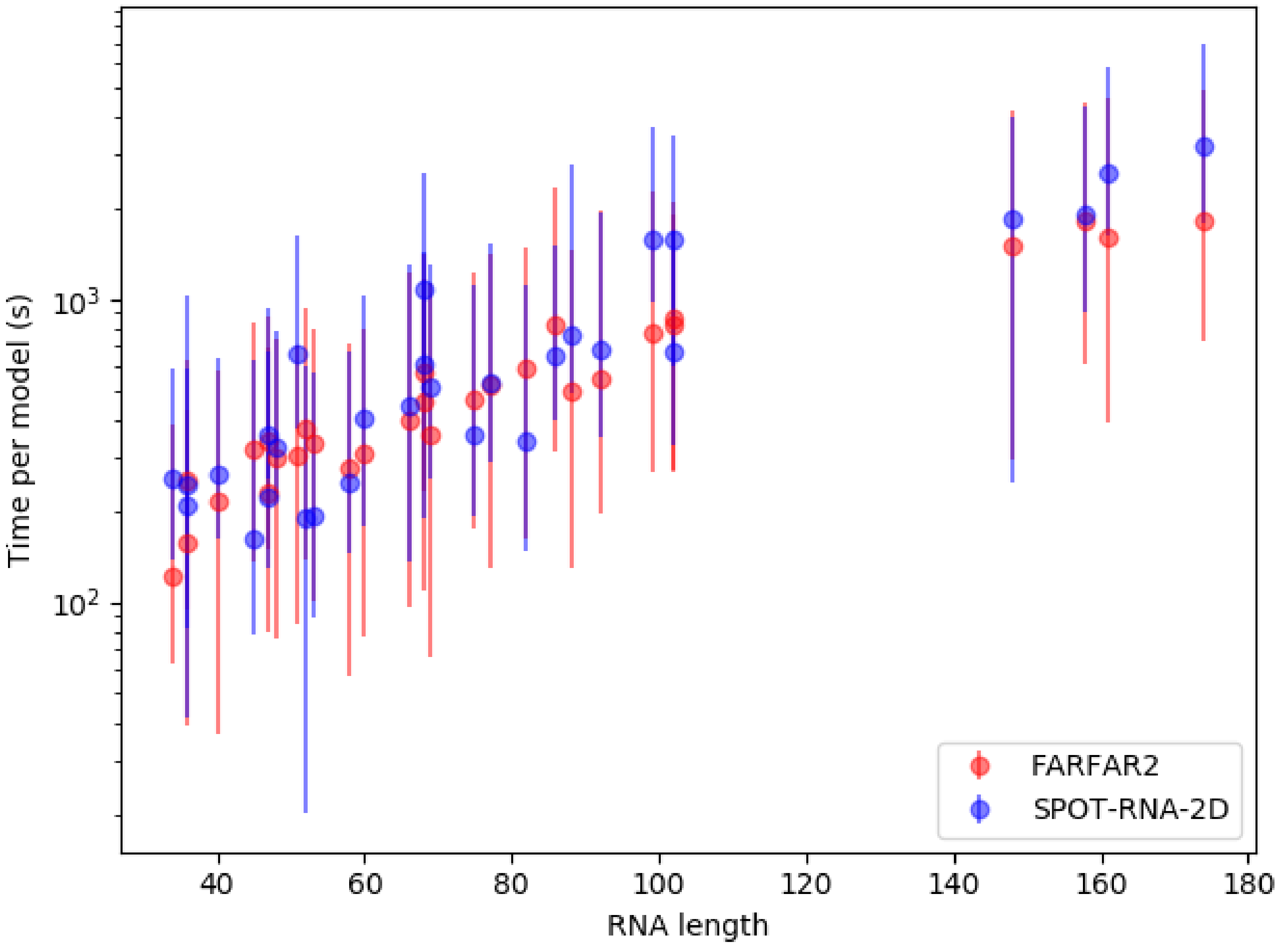

Figure S5. Time taken to generate a single model for each target in the TS2 dataset by FARFAR2 and SPOT-RNA-2D given the relevant inputs have been pre-generated. Error bars indicate the upper and lower quartile.

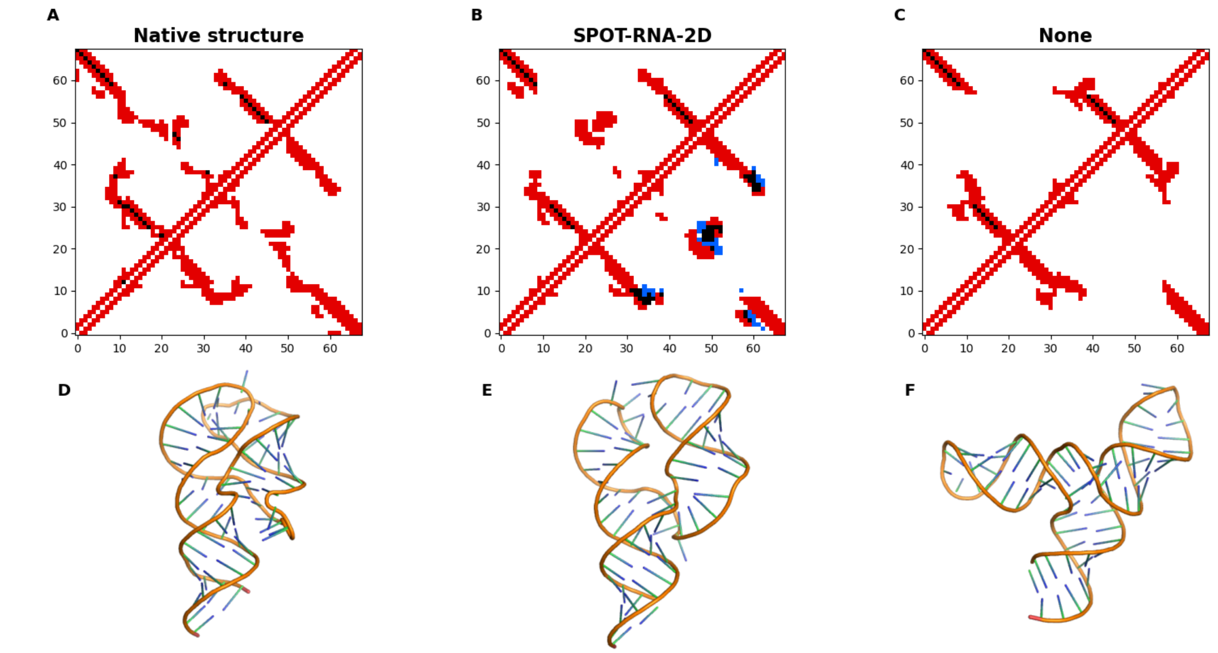

Figure S6. *First row:* Distance-derived contact maps for 2'-dG riboswitch from the **(A)** Native structure, **(B)** Model with SPOT-RNA-2D constraints, **(C)** Model with no tertiary constraints. *Second row:* 3D structure representation of 2'-dG riboswitch from **(D)** Chain A, PDB ID: 3slm, **(E)** top 1 model with SPOT-RNA-2D constraints and **(F)** top 1 FARFAR2 model

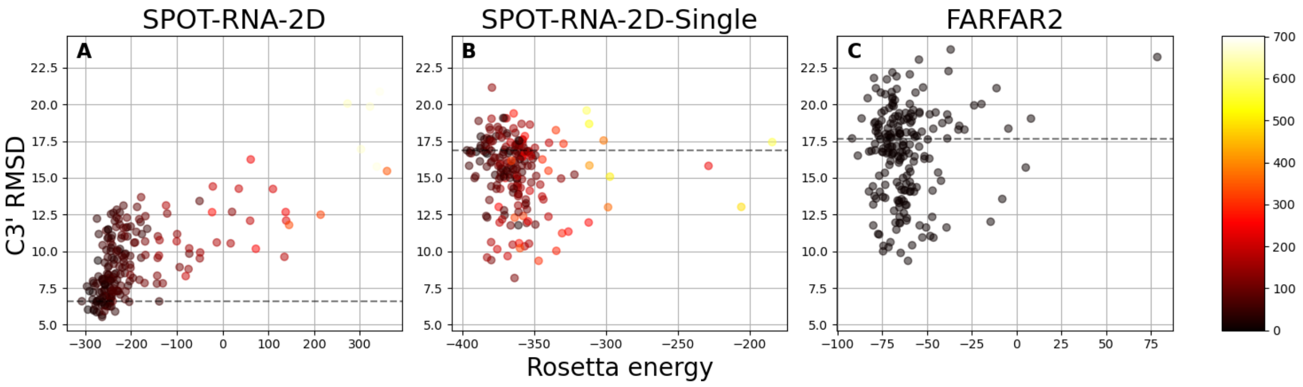

Figure S7. Plot of Rosetta energy against model C3' RMSD for all 200 models of the 3slm_A target by (A) SPOT-RNA-2D, (B) SPOT-RNA-2D-Single and (C) FARFAR2. The color gradient illustrates the contribution of the contact map constraints to the energy score
